# First‐in‐Asian Phase I Study of the Anti‐Fibroblast Growth Factor 23 Monoclonal Antibody, Burosumab: Safety and Pharmacodynamics in Adults With X‐linked Hypophosphatemia

**DOI:** 10.1002/jbm4.10074

**Published:** 2018-09-14

**Authors:** Hae Il Cheong, Han‐Wook Yoo, Masanori Adachi, Hiroyuki Tanaka, Ikuma Fujiwara, Yukihiro Hasegawa, Daisuke Harada, Maiko Sugimoto, Yosuke Okada, Masaki Kato, Ryutaro Shimazaki, Keiichi Ozono, Yoshiki Seino

**Affiliations:** ^1^ Department of Pediatrics Seoul National University Children's Hospital Seoul Republic of Korea; ^2^ Department of Pediatrics Asan Medical Center Seoul Republic of Korea; ^3^ Department of Endocrinology and Metabolism Kanagawa Children's Medical Center Kanagawa Japan; ^4^ Department of Pediatrics Okayama Saiseikai General Hospital Okayama Japan; ^5^ Department of Pediatric Endocrinology and Environmental Medicine Tohoku University Graduate School of Medicine Miyagi Japan; ^6^ Division of Endocrinology and Metabolism Tokyo Metropolitan Children's Medical Center Tokyo Japan; ^7^ Department of Pediatrics Osaka Hospital Japan Community Healthcare Organization (JCHO) Osaka Japan; ^8^ Kyowa Hakko Kirin Co. Ltd Tokyo Japan; ^9^ Department of Pediatrics Osaka University Graduate School of Medicine Osaka Japan

**Keywords:** CELL/TISSUE SIGNALING—ENDOCRINE PATHWAYS, PTH/VIT D/FGF2, CLINICAL TRIALS, DISEASES AND DISORDERS OF/RELATED TO BONE, OSTEOMALACIA AND RICKETS, DISORDERS OF CALCIUM/PHOSPHATE, OTHER

## Abstract

X‐linked hypophosphatemia (XLH) is a disease caused by abnormally elevated FGF23 levels, which cause persistent hypophosphatemia accompanied by subsequent reduction in bone mineralization that presents as rickets or osteomalacia. Burosumab is a fully human monoclonal antibody targeting FGF23 that is under development for the treatment of FGF23‐related hypophosphatemia including XLH. The safety, tolerability, and proof of concept of burosumab have been evaluated in patients with XLH in previous studies conducted in countries outside of Asia. The objective of this study was to evaluate the safety, tolerability, pharmacokinetics (PK), pharmacodynamics (PD), and expression of anti‐drug antibodies in Japanese and Korean adults with XLH. This was a multicenter, sequential dose‐escalation, open‐label, single‐dose study. This study began with cohort 1 (s.c. dose of burosumab 0.3 mg/kg), after which the dose was escalated sequentially in cohort 2 (s.c. dose of burosumab 0.6 mg/kg) and cohort 3 (s.c. dose of burosumab 1.0 mg/kg). The PK of burosumab were linear within the dose range of 0.3 to 1.0 mg/kg. The PD effects such as serum phosphorus concentration, serum 1,25[OH]_2_D_3_ concentration, and ratio of tubular maximum reabsorption rate of phosphate to glomerular filtration rate (TmP/GFR) were elevated after a single s.c. administration. The area under the receiver‐operating characteristic curve from 0 to *t* (AUC_0–t_) values calculated using the change from baseline values of serum phosphorus, serum 1,25(OH)_2_D_3_, and TmP/GFR were correlated with the AUC_0–t_ of burosumab. Furthermore, no serious adverse events (AEs), deaths, remarkable increase or decrease in the corrected calcium or intact PTH levels, or signs of nephrocalcinosis or its worsening were observed after treatment. Some AEs and drug‐related AEs were observed; however, there were no clinically meaningful tendencies. The positive effects and acceptable safety profile seen in this study are encouraging for Japanese and Korean patients with XLH. © 2018 The Authors *JBMR Plus* published by Wiley Periodicals, Inc. on behalf of American Society for Bone and Mineral Research.

## Introduction

X‐linked hypophosphatemia (XLH) is a congenital disease characterized by high levels of circulating FGF23, which are caused by loss‐of‐function mutations in the *PHEX* gene.^1^ FGF23 decreases phosphorus (P) reabsorption in the kidney by decreasing the expression of the sodium‐dependent inorganic phosphate transporter type IIa/c (NaPi‐IIa/c) in the renal proximal tubule.^2^ Furthermore, FGF23 decreases serum 1,25[OH]_2_D_3_ levels by suppressing 1α‐hydroxylase expression and promoting the expression of 24‐hydroxylase, and it decreases intestinal absorption of P.^3^, ^4^ FGF23 plays an important role in maintaining phosphate homeostasis by these mechanisms, and its production adjusts according to serum P levels.^5^, ^6^ In XLH, excess FGF23 leads to a decrease in serum P levels below the normal range, resulting in deficient bone mineralization from the early postnatal period.^3^, ^4^ As a consequence, bowed long bones such as knock‐knee, genu valgum, and ossification disorder of the growth plate occur,^1^, ^7^ and normal growth is impaired in childhood. Patients with XLH are known to have an abnormal gait and bone pain due to bone deformity and loading, respectively, no age‐appropriate exercise capacity, and frequent dental abscesses.^1^ Because hypophosphatemia, osteomalacia, and lower‐limb deformity persist in adulthood, patients can have bone pain, increased fracture or microfracture risk, dysarthrosis, arthralgia, muscular weakness, and enthesopathy, which affect walking, working, or both.^1^, ^8^, ^9^, ^10^ Therefore, XLH markedly decreases the overall lifetime quality of life (QOL).^(1,11)^ The current therapy for XLH is supplemental treatment with oral phosphate and active vitamin D formulation. Because of high FGF23 levels, excessive urinary phosphate excretion, and decreased vitamin D activation, frequent administration of high‐dose oral phosphate is required to maintain serum P levels close to the normal range.^1^, ^12^ However, taking oral phosphate frequently and at a high dose may lead to gastrointestinal symptoms such as abdominal pain and diarrhea or, in the case of long‐term use, secondary hyperparathyroidism.^13^ Similarly, administration of excess active vitamin D formulations causes hypercalcemia, hypercalciuria, or both, which can result in ectopic, mainly renal, calcification.^13^ The dosage of oral phosphate and active vitamin D formulation is currently limited because of concern about possible adverse reactions, making it difficult to maintain serum P levels within the normal range preferred for treatment.^7^, ^11^ Therefore, the current therapy has limited beneficial effects in improving the final height, bone symptoms, or both, and adverse reactions including renal calcification, secondary hyperparathyroidism, and ossification of ligaments occur with long‐term use despite the limited dose.^11^, ^13^ Consequently, a new treatment strategy is required that would not lead to rapid changes in serum P and calcium (Ca) levels or cause adverse events (AEs) associated with current therapy and that can maintain serum P levels within a normal range.

Burosumab is a recombinant fully human monoclonal antibody (immunoglobulin G1) that directly binds to FGF23 and blocks the intracellular signaling caused by the FGF23/Klotho/FGF receptor 1 (FGFR1) complex.^14^ Burosumab neutralizes the action of FGF23 that causes hypophosphatemia in XLH and thereby normalizes P reabsorption in the kidney.^15^, ^16^ Furthermore, its intestinal absorption is associated with increased 1,25(OH)_2_D_3_ levels, which may improve chronic hypophosphatemia.^15^, ^16^

Clinical studies conducted in the United States and Canada have evaluated the safety and tolerability of single and repeated (28‐day intervals) s.c. doses of burosumab up to 1.0 mg/kg in adult patients with XLH.^15^, ^16^ The studies demonstrated that burosumab continuously increased serum P levels and the ratio of tubular maximum reabsorption rate of phosphate to glomerular filtration rate (TmP/GFR) without significantly affecting the urinary excretion of Ca or vitamin D metabolism, thus confirming the proof of concept of burosumab in patients with XLH.^15^, ^16^ However, burosumab has not been administered to Asian patients, and its safety and tolerability in these patients are unknown.

Therefore, in this study, we evaluated the safety and tolerability of single s.c. doses of burosumab 0.3, 0.6, or 1.0 mg/kg in Japanese and Korean patients with XLH. We also evaluated the pharmacokinetics (PK), pharmacodynamics (PD), and expression of anti‐drug antibodies as secondary endpoints.

## Patients and Methods

### Patients

Japanese and Korean patients were enrolled after providing their informed consent. The key inclusion criteria were age ≥18 years and diagnosis of XLH. The key exclusion criteria included: active infection or chronic inflammatory disease; uncontrolled hypertension; uncontrolled diabetes mellitus; history of known immunodeficiency; use of a pharmacological vitamin D metabolite or its analogs within 21 days prior to and after screening; use of phosphate, Ca preparation, calcimimetics, aluminum hydroxide antacids, thiazide diuretic, acetazolamide, or phosphate‐, Ca‐, and/or vitamin D‐containing supplements within 10 days prior to and after screening; and women who were pregnant, possibly pregnant, lactating, or who had no intention of using adequate contraception.

### Study design

**Figure 1:**
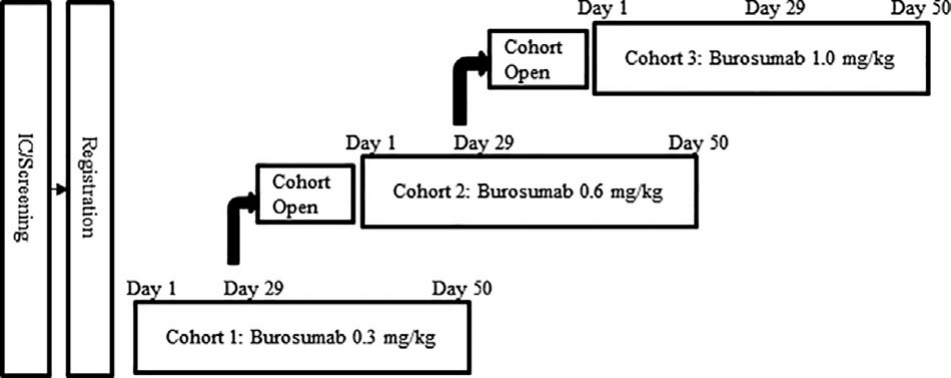
Schematic study design. This was a sequential dose‐escalation, open‐label, single‐dose study. After the fifth patient in each cohort completed the day 29 visit, safety data were reviewed to determine whether it was appropriate to commence enrollment for the next cohort. The target number of patients was five per cohort (15 in total). IC = informed consent.

This was a multicenter, sequential dose‐escalation, open‐label, single‐dose study (Fig. 1). The investigators screened the patients after obtaining their informed consent. Eligible patients were enrolled and received a single s.c. dose of burosumab on day 1. Cohort 1 received burosumab 0.3 mg/kg, cohort 2 received burosumab 0.6 mg/kg, and cohort 3 received burosumab 1.0 mg/kg. After the fifth patient in each cohort was examined on day 29, the data monitoring committee reviewed the safety data and determined whether it was appropriate to advance to the next cohort.

### Study assessments

The following measurements were taken in each patient: height and weight, vital signs, hematology, blood chemistry (including P, 1,25[OH]_2_D_3_, intact PTH [iPTH], corrected serum Ca, creatinine [Cr], serum 25OHD, calcitonin, and alkaline phosphatase [ALP]), urinalysis, 2‐hour urinary parameters (urinary P, Ca, and Cr), 24‐hour urine analysis (urinary P, Ca, and Cr), 12‐lead electrocardiography (ECG), renal ultrasound (graded from grade 0 to grade 4), chest X‐ray, pregnancy test (except for patients with no childbearing potential), serology, intact FGF23 (iFGF23), serum FGF23 concentrations, serum burosumab concentration, and anti‐drug antibodies. AEs were categorized by relationship to burosumab treatment.

### Sample analysis

Measurement of 1,25(OH)_2_D_3_, iPTH, 25OHD, calcitonin, 2‐hour and 24‐hour urine collections, and iFGF23 was performed by SRL Medisearch Inc (Tokyo, Japan). Serum burosumab, serum FGF23, and anti‐drug antibodies were measured by Toray Research Center, Inc. (Kanagawa, Japan). Serum burosumab concentrations were measured using a validated sandwich electrochemiluminescence (ECL)‐based ligand binding assay (data on file), which used an anti‐drug idiotype capture antibody and a ruthenylated anti‐drug detection antibody (both manufactured by Kyowa Hakko Kirin Co., Ltd., Tokyo, Japan). ECL signals were measured, and burosumab concentrations were calculated. The calibration range was 50 to 10,000 ng/mL, and the lower limit of quantification was 50 ng/mL. The assay accuracy (relative error) and precision (coefficient of variation for the mean) were within the acceptable range (< ±20%). The relative error of 10 individual human serum samples spiked with 50 ng/mL burosumab was within the acceptable range (< ±25%), whereas all unspiked individual samples yielded concentrations below the lower limit of quantification (50 ng/mL), confirming the selectivity of the assay. The anti‐drug antibodies were assayed using a validated ECL‐based ligand‐binding assay. A sequential screening algorithm was used as an initial screening assay, and subsequent immunodepletion and neutralizing assays were performed. Testing only proceeded to the subsequent assay step if the earlier assay was positive.

### Study approval

This study was conducted in accordance with the principles of the Declaration of Helsinki, and in compliance with the standards and guidelines of Japan and Korea. This study was approved by the institutional review boards of each participating institution. Written informed consent was received from all patients prior to their inclusion.

## Results

### Patient characteristics

**Figure 2:**
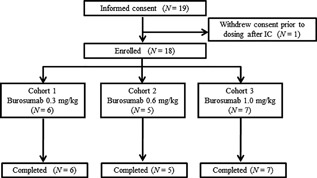
Study flow diagram. Eighteen adult patients with XLH were enrolled, consisting of six, five, and seven patients in cohorts 1, 2, and 3, respectively (administered burosumab 0.3, 0.6, and 1.0 mg/kg, respectively). No patient withdrew after administration, and all enrolled patients underwent complete follow‐up and analysis. IC = informed consent.

Eighteen adult patients with XLH were enrolled in the study, consisting of six, five, and seven patients in cohorts 1, 2, and 3, respectively (Table 1, Fig. 2). No patient withdrew after administration of burosumab, and all enrolled patients underwent a complete follow‐up and analysis. Prior to dosing, the median (minimum–maximum) serum P concentration was 1.85 (1.0–2.4) mg/dL. The median TmP/GFR was 1.56 (0.6–2.0) mg/dL, and all values were below the lower limit of the reference range.^9^ The median serum 1,25(OH)_2_D_3_ concentration was 52.65 (11.8–71.9) pg/mL. In contrast, the iFGF23 concentrations were highly variable and ranged from 36.9 to 1260.0 pg/mL across cohorts.

**Table 1:** Baseline Patient Demographics and Disease Characteristics

Characteristic	Overall	Cohort 1 (0.3 mg/kg)	Cohort 2 (0.6 mg/kg)	Cohort 3 (1.0 mg/kg)
Patients, *n*	18	6	5	7
Age (year)	34.6 (19–57)	37.3 (21–49)	31.6 (19–49)	34.4 (19–57)
Sex, male/female, *n*	6/12	1/5	1/4	4/3
Nationality, Japanese/Korean, *n*	10/8	3/3	3/2	4/3
Weight (kg)	58.99 (36.7–89.9)	53.08 (36.7–64.8)	61.70 (53.1–89.9)	62.11 (52.8–84.5)
Height (cm)	147.62 (117.6–167.6)	144.83 (126.2–159.0)	154.92 (146.6–167.6)	144.79 (117.6–158.6)
Serum P (mg/dL)	1.77 (1.0–2.4)	1.80 (1.4–2.1)	1.92 (1.4–2.4)	1.63 (1.0–2.1)
Serum 1,25(OH)_2_D_3_ (pg/mL)	47.99 (11.8–71.9)	53.90 (11.8–71.9)	48.56 (29.3–60.7)	42.53 (17.8–71.4)
Serum 25OHD (ng/mL)	17.8 (11–28)	19.2 (14–28)	19.2 (13–24)	15.7 (11–23)
Serum Ca (mg/dL)	9.36 (8.6–10.4)	9.42 (8.6–10.4)	9.32 (8.8–9.7)	9.34 (8.8–9.7)
Serum Cr (mg/dL)	0.569 (0.40–1.02)	0.618 (0.48–1.02)	0.552 (0.43–0.62)	0.539 (0.40–0.80)
Serum iPTH (pg/mL)	90.4 (28–155)	87.3 (28–155)	86.0 (58–145)	96.3 (64–145)
Serum intact FGF23 (pg/mL)	213.57 (36.9–1260.0)	272.00 (36.9–1260.0)	105.76 (59.4–221.0)	240.50 (41.7–997.0)
TmP/GFR (mg/dL)	1.51 (0.6–2.0)	1.50 (1.1–1.7)	1.71 (1.1–2.0)	1.38 (0.6–2.0)
24‐Hour urine Ca (mg)	0.097 (0.03–0.28)	0.080 (0.03–0.16)	0.086 (0.04–0.19)	0.119 (0.04–0.28)
2‐Hour urine Ca/Cr (mg/g)	51.27 (0.0–110.1)	53.73 (10.4–103.4)	38.98 (0.0–64.4)	57.94 (23.6–110.1)

Values are mean (minimum–maximum) unless indicated otherwise.

P = phosphorus; Ca = calcium; Cr = creatinine; iPTH = intact PTH; TmP/GFR = tubular maximum reabsorption rate of phosphate to glomerular filtration rate.

### PK analysis

**Figure 3:**
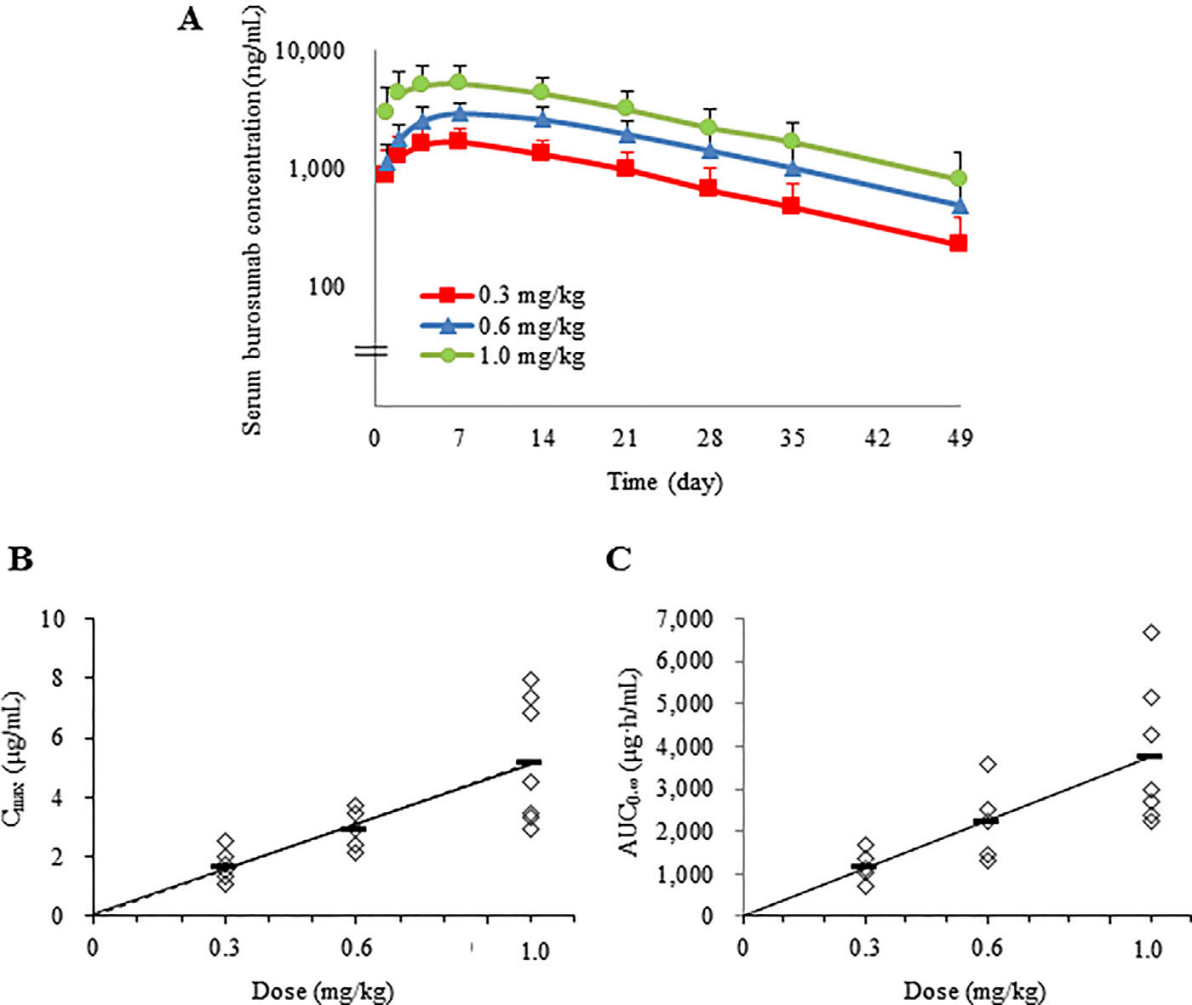
Burosumab PK profiles following single‐dose administration of burosumab 0.3, 0.6, and 1.0 mg/kg. (*A*) Serum burosumab concentration–time profile. Data are mean + SD. Dose dependence of (*B*) C_max_ and (*C*) AUC_0–∞_ in individual patients (diamond symbols). Solid and broken lines represent the linear regression line with and without the intercept, respectively. C_max_ = maximum drug concentration; AUC_0–∞_ = area under the receiver‐operating characteristic curve from 0 to infinity.

After a single s.c. administration of burosumab at doses of 0.3, 0.6, or 1.0 mg/kg, the serum burosumab concentrations increased dose‐dependently (Fig. 3 *A*). The median time to achieve the maximum drug concentration (T_max_) was 166 or 167 hours for all three doses (Table 2). Serum burosumab was eliminated with a mean ± SD t_*1/2*_ of 289 ± 121 hours in cohort 1, 315 ± 131 hours in cohort 2, and 336 ± 85 hours in cohort 3, which was approximately equivalent to 12 to 14 days (Table 2). The maximum drug concentration (C_max_) and area under the receiver‐operating characteristic curve from 0 to infinity (AUC_0–∞_) increased dose‐dependently in the dose range 0.3 to 1.0 mg/kg (Table 2; Fig. 3 *B*, *C*).

**Table 2:** Summary of Burosumab PK Parameters

Cohort	Dose (mg/kg)	*n*	t_max_ (hours)	C_max_ (μg/mL)	AUC_0–__∞_ (μg/h/mL)	t_1/2_ (hours)
1	0.3	6	166 (46.5–168)	1.71 ± 0.51	1180 ± 370^a^	289 ± 121^a^
2	0.6	5	167 (165–334)	2.95 ± 0.67	2220 ± 920	315 ± 131
3	1.0	7	166 (93.5–168)	5.19 ± 2.12	3770 ± 1670	336 ± 85

t_max_ is shown as median (minimum–maximum), and other PK parameters are shown as mean ± SD.

PK = pharmacokinetic; *n* = number of subjects used for descriptive statistics of each PK parameter; t_max_ = time to maximum drug concentration; C_max_ = maximum drug concentration; AUC_0–∞_ = area under the receiver‐operating characteristic curve from 0 to infinity.

^a^ *n* = 5.

### Serum P, serum 1,25(OH)_2_D_3_, and TmP/GFR

**Figure 4:**
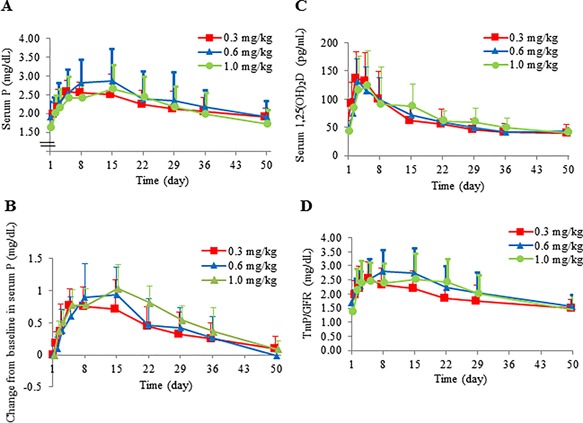
Effect of burosumab administration on pharmacodynamic parameters. Concentration–time profiles of (*A*) serum P, (*C*) 1,25(OH)_2_D_3_, and (*D*) TmP/GFR. (*B*) Change from baseline in serum P. Data are mean + SD. P = phosphorus; TmP/GFR = tubular maximum reabsorption rate of phosphate to glomerular filtration rate.

Serum P concentrations, serum 1,25(OH)_2_D_3_ concentrations, and TmP/GFR increased after burosumab administration in all cohorts (Fig. 4). The maximum (mean ± SD) concentration of serum P occurred between day 5 and day 15 and was 2.57 ± 0.31 mg/dL on day 5 in cohort 1, 2.86 ± 0.85 mg/dL on day 15 in cohort 2, and 2.66 ± 0.63 mg/dL on day 15 in cohort 3 (Fig. 4 *A*). Although no clear dose‐response relationship was observed, the maximum serum P concentrations were higher in cohorts 2 and 3 than in cohort 1, and the maximum change from baseline increased dose‐dependently (Fig. 4 *B*). The maximum 1,25(OH)_2_D_3_ concentration occurred between day 3 and day 5 and was 137.58 ± 46.30 pg/mL on day 3 in cohort 1, 133.00 ± 38.18 pg/mL on day 3 in cohort 2, and 123.04 ± 62.21 pg/mL on day 5 in cohort 3 (Fig. 4 *C*). The maximum TmP/GFR values occurred between day 5 and day 15 and were 2.5637 ± 0.5637 mg/dL on day 5 in cohort 1, 2.7950 ± 0.7599 mg/dL on day 8 in cohort 2, and 2.6196 ± 0.8064 mg/dL on day 15 in cohort 3 (Fig. 4 *D*). After reaching maximum levels, serum P, serum 1,25(OH)_2_D_3_, and TmP/GFR gradually decreased, returning to approximately baseline levels by day 50 (Fig. 4).

### Other biochemical outcomes

**Figure 5:**
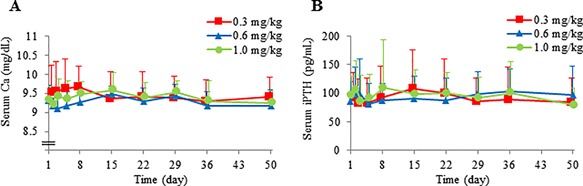
Effect of burosumab administration on serum (*A*) Ca and (*B*) iPTH. Data are mean + SD. Ca = calcium; iPTH = intact PTH.

Corrected Ca and iPTH concentrations did not indicate a clear increase or decrease after administration of burosumab (Fig. 5 *A*, *B*, respectively). There was also no clear change in the urinary Ca concentration, total urinary Ca, 25OHD, and other parameters (data not shown).

### PK/PD analysis

**Figure 6:**
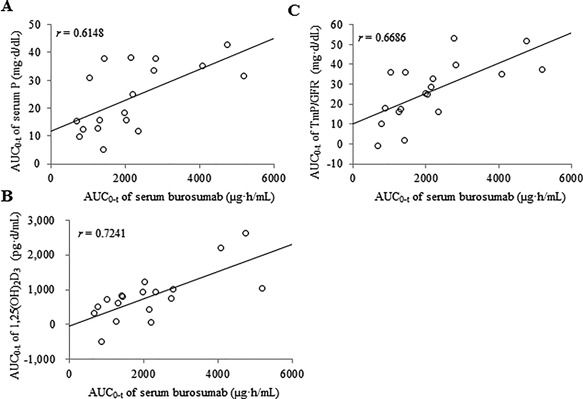
Relationship between PK and PD parameters. Scatter plot of AUC_0–t_ of serum burosumab versus AUC_0–t_ of (*A*) serum P, (*B*) 1,25[OH]_2_D_3_, and (*C*) ratio of renal TmP/GFR. AUC_0–t_ = area under the receiver‐operating characteristic curve from 0 to time *t*; P = phosphorus; TmP/GFR = tubular maximum reabsorption rate of phosphate to glomerular filtration rate.

The AUC from 0 to *t* (AUC_0–t_) of serum P, serum 1,25(OH)_2_D_3_, and TmP/GFR increased with increasing AUC_0–t_ of serum burosumab, with *r* values of 0.6148, 0.7241, and 0.6686, respectively (Fig. 6 *A–C*).

### Safety

Overall, 14 of the 18 patients (78%) experienced AEs, as follows: four of six patients (67%) in cohort 1, four of five patients (80%) in cohort 2, and six of seven patients (86%) in cohort 3. The most common AEs were nasopharyngitis (five patients, 28%), upper respiratory tract infection (four patients, 22%), contusion (three patients, 17%), headache (three patients, 17%), and back pain (two patients, 11%). Overall, five of the 18 patients (28%) experienced drug‐related AEs, as follows: three of six patients (50%) in cohort 1 and two of seven patients (29%) in cohort 3; no drug‐related AEs occurred in cohort 2. The reported drug‐related AEs were nausea, feeling hot, pyrexia, back pain, abnormal white blood cell count, and headache, in one patient each. All AEs and drug‐related AEs were grade 1 (mild) or grade 2 (moderate). In the laboratory measurements, elevations related to the PD effects were observed. No other clinically meaningful changes were observed in the mean values. Vital signs showed slight changes from baseline in all patients, but no clear tendencies were observed. In the 12‐lead ECG, renal ultrasound, and chest X‐ray, no shifts from normal to abnormal levels after treatment were observed. Furthermore, no signs of nephrocalcinosis or its worsening were observed after treatment. One patient in cohort 1 tested positive for anti‐drug binding antibodies before treatment and on day 50 but was negative for anti‐drug neutralizing antibodies at both time points. No AEs were reported in patients who were positive for anti‐drug binding antibodies. No serious AEs, deaths, AEs leading to study discontinuation, or other significant AEs were observed. Therefore, the safety and tolerability of burosumab after a single s.c. administration at up to 1.0 mg/kg in patients with XLH in Japan and Korea was evaluated and showed promise.

## Discussion

In this study, burosumab was administered for the first time to Japanese and Korean patients with XLH, and we confirmed that serum burosumab levels increased dose dependently. In all patients, serum P levels, 1,25(OH)_2_D_3_ levels, and TmP/GFR increased. After burosumab administration, serum P levels peaked on day 5 to day 15 and never exceeded the upper limit of normal (4.5 mg/dL). These findings suggest that phosphate wasting is improved by neutralizing the effect of FGF23. No remarkable increase or decrease in the corrected Ca or iPTH levels was observed, and no other clinically significant AEs occurred.

Although the PK data in this study differed slightly from those obtained in a single‐dose study in the United States,^15^ the differences were minor. Furthermore, no differences were observed in patient demographics such as weight and body mass index, as well as serum P or iFGF23 levels, between this study and a single‐dose study in the United States.^15^ Therefore, the differences may be attributed to the limited sample size in this study and intraindividual variability.^15^ The PD markers in this study, such as serum P, 1,25(OH)_2_D_3_, TmP/GFR, Ca, and iPTH levels, were not markedly different from those obtained in the single‐dose study in the United States.^15^ No AEs characteristic of Asians occurred, and these findings indicate that there was no marked racial difference in the PK, PD, or safety of burosumab.

The clinical study of burosumab administered as a repeated s.c. dose every 28 days in the United States and Canada has shown that burosumab continuously improved serum P levels without causing rapid changes in the levels and did not change serum Ca levels significantly.^(16)^ Given that this first‐in‐Asian phase I study indicated no marked racial difference in the PK, PD, and safety of a single dose of burosumab, the results suggest that repeated doses of burosumab may sustain serum P levels without rapid changes and without changing serum Ca levels in Japanese and Korean patients, as has been shown in patients in the United States and Canada.

These findings suggest that adverse reactions such as renal calcification and secondary hyperparathyroidism, resulting from rapid changes in serum P and Ca levels, are unlikely to occur with burosumab treatment. Furthermore, it is anticipated that burosumab will normalize serum P levels continuously, and because burosumab improves phosphate wasting, treatment may improve osteomalacia‐related symptoms, physical function, and QOL. However, evaluation of the safety profile in this study is constrained by the short duration of assessment and the limited endpoints of PD markers. Further verification of the safety and efficacy of burosumab is required, including an evaluation of its long‐term safety and the establishment of appropriate endpoints for osteomalacia‐related symptoms, physical function, and QOL.

In conclusion, this study confirmed that burosumab increased serum P levels, 1,25(OH)_2_D_3_ levels, and TmP/GFR in Japanese and Korean patients with XLH. The positive effects and acceptable safety profile observed in this burosumab study are encouraging for Japanese and Korean patients with XLH.

## Disclosures

KO and YS have received consulting fees from Kyowa Hakko Kirin Co., Ltd. All other authors state that they have no conflicts of interest.
